# Adjusting for 30-hour undigested neutral detergent fiber in substitution of wheat straw and beet pulp for alfalfa hay and corn silage in the dairy cow diet: Chewing activities, diurnal feed intake, and ruminal fermentation

**DOI:** 10.3168/jdsc.2022-0248

**Published:** 2022-09-03

**Authors:** Ali Kahyani, Gholam Reza Ghorbani, Masoud Alikhani, Hassan Rafiee, Omid Ramezani, Mehdi Asemi Esfahani, Farhad Ahmadi

**Affiliations:** 1 Department of Animal Sciences, College of Agriculture, Isfahan University of Technology, Isfahan 84156-83111, Iran; 2 Animal Science Research Department; Isfahan Agriculture and Natural Resources Research and Education Center; Agriculture Research, Education and Extension Organization (AREEO), 8174835117, Isfahan, Iran; 3 Department of Animal Science, Campus of Agriculture and Natural Resources, University of Tehran, 31587-77871, Karaj, Iran; 4 Department of Animal Science, Agricultural Sciences and Natural Resources, University of Khuzestan, 63417-73637, Mollasani, Ahvaz, Iran; 5 Department of Eco-friendly Livestock Science, Institute of Green Bio Science and Technology, Seoul National University, Pyeongchang, 25354, South Korea

## Abstract

This experiment investigated the effects of replacing alfalfa hay (AH) and corn silage (CS) with wheat straw (WS) and beet pulp (BP) in diets with similar forage 30-h undigested neutral detergent fiber (uNDF_30_) on chewing behavior and ruminal fermentation of lactating cows. Twelve multiparous Holstein cows (51 ± 3 kg/d of milk; days in milk = 97 ± 13; mean ± standard error) were housed in individual stalls and used in a replicated 3 × 3 Latin square design with 28-d periods. Experimental diets were (1) 0% forage uNDF_30_ from WS (WS0, contained 2% BP); (2) 50% forage uNDF_30_ from WS (WS50, contained 7.6% BP); and (3) 100% forage uNDF_30_ from WS (WS100, contained 12.4% BP). From 0 to 2 h after the morning feeding, there was a tendency observed for a quadratic effect on dry matter intake (DMI), with cows fed WS50 consuming the greatest amount of DM (9.19 kg). Later DMI (4 to 6 h and 6 to 24 h postfeeding) decreased as dietary proportion of WS and BP increased. Increasing WS and BP decreased eating behavior, but had no detected effect on rumination time (455 min/d), which resulted in a linear reduction in chewing time (the sum of eating and rumination activities). As WS and BP inclusion increased, the number of meals decreased linearly, whereas time between meals, eating rate, and meal size per kilogram of DM increased linearly. Increased dietary inclusion of WS and BP tended to decrease total ruminal VFA and resulted in a linear decrease in propionate but an increase in acetate proportion and ammonia-N concentration in the rumen. Overall, the substitution had no effect on rumination activity, possibly suggesting that a combination of WS and BP could be used in dairy cow rations as substitutes for high-quality forages when WS was added to maintain the uNDF_30_ level.

## Highlights

• WS and BP were substituted for corn silage and alfalfa hay in diets with similar forage undigested NDF and dietary NDF digestibility.
• As the proportion of WS and BP increased in the diet, time spent eating decreased.
• As the proportion of WS and BP increased in the diet, acetate:propionate and ammonia-N concentration in the rumen increased.

## Graphical Abstract

**Figure:**
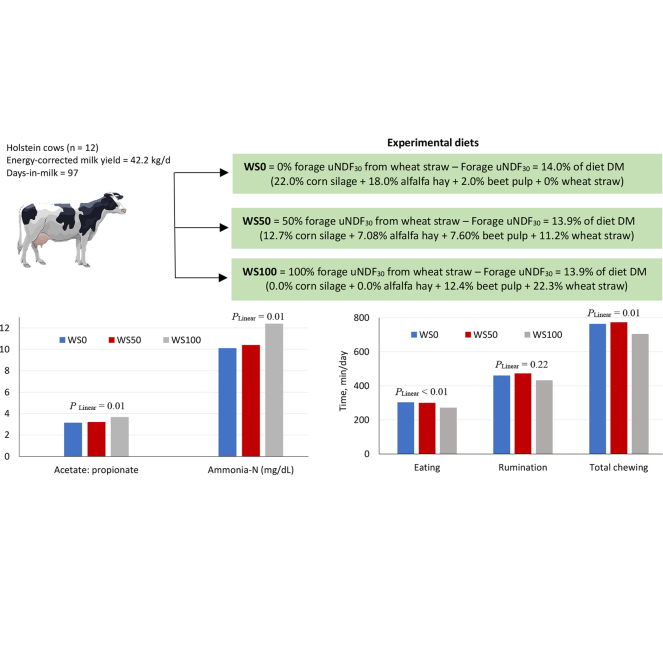
**Summary:** Feeding behavior and ruminal fermentation characteristics of lactating cows were investigated when offered rations supplying similar forage undigested neutral detergent fiber (NDF) and dietary NDF digestibility (both measured after 30 hours of in situ incubation). Diets contained wheat straw (WS) and beet pulp (BP), which replaced corn silage and alfalfa hay. Time spent eating decreased linearly and rumination activity remained unaffected as the proportion of WS and BP increased. This resulted in a linear reduction in chewing behavior.

Providing adequate fiber to dairy cows is critical for maintaining rumen function and chewing behavior. Intake of dietary forage is important as it primarily contributes to the physically effective NDF (**peNDF**) portion of the diet, which is responsible for stimulating rumination and improving fiber digestibility (Zebeli et al., 2012). Corn silage (**CS**) and alfalfa hay (**AH**) are 2 main forage sources commonly used in dairy cow diets. However, these forages are sometimes limited, and low-quality forages such as wheat straw (**WS**) are occasionally used in dairy rations. Wheat straw is usually used in dairy cow rations as a peNDF source to promote rumination (Eastridge et al., 2009). Poor fiber digestibility in low-quality forages such as WS is associated with a longer retention time in the rumen and a slower outflow, which may decrease DMI (Oba and Allen, 1999) and compromise milk production of high-producing dairy cows.

Feeding and rumination behavior, ruminal fill, DMI, and milk production could be influenced by neutral detergent fiber digestibility (**NDFD**). Forage NDF digestibility has a large impact on rumen turnover and fill, and thus voluntary feed intake and performance, particularly in high-producing cows (Oba and Allen, 1999; Mertens, 2016). We theorized that in dairy cow rations formulated to supply a similar forage 30-h undigested neutral detergent fiber (**uNDF_30_**) and dietary NDFD_30_, a combination of WS, a low-quality forage source, and a highly digestible source of fiber such as beet pulp (**BP**; in vitro NDFD of 76–90% after 30 and 48 h incubations; Hoffman and Combs, 2003) could replace high-quality forages without affecting diurnal feed intake patterns, chewing activities, or ruminal fermentation in high-producing cows. Furthermore, the use of BP with WS can partially compensate for the dietary energy deficiency without increasing cereal grain or starch level, which may negatively affect fiber digestibility (Firkins, 1997). However, nonforage fiber sources do not stimulate chewing activity due to their small particle size and high digestibility (Zebeli et al., 2012).

Kahyani et al. (2019) reported that WS, BP, CS, and AH differ widely in the content of undigested NDF (at 30, 240, and 288 h of incubation), NDFD, as well as the digestion rate of potentially digestible neutral detergent fiber fraction (**pdNDF**). Regardless of NDF content, CS typically contains a higher proportion of pdNDF with a lower digestion rate than AH (Van Soest, 1994). Moreover, WS has a lower concentration of pdNDF with a slower rate of digestion than BP (Kahyani et al., 2019). Therefore, formulating dairy rations on the basis of NDF concentration alone would result in diets having variable content of pdNDF, uNDF, and digestion rates.

The simultaneous replacement of CS and AH with WS and BP is a nutritional strategy that could maintain feed intake and productivity of high-producing dairy cows (Kahyani et al., 2019). However, their effects on feeding behavior and ruminal fermentation characteristics are not clearly understood. Therefore, the objective of this study was to evaluate the substitution effect of WS and BP for AH and CS with fixed forage uNDF_30_ and dietary NDFD_30_ on feeding behavior and ruminal fermentation of high-producing dairy cows.

The Institutional Animal Care Committee for Animals Used in Research approved the experimental protocols. Care for animals was based on guidelines set by the Iranian Council for Animal Care (1995). This research is a continuation of a larger project that was performed using 12 multiparous Holstein cows and data on DMI, lactation performance, and nutrient digestibility were previously reported (Kahyani et al., 2019). In brief, cows were housed in individual 4 × 4 m stalls, and the total amount of feed was offered twice daily (1000 and 1800 h) and adjusted daily to ensure approximately 10% feed refusal per cow. Actual feed refusal averaged 9.75 ± 3.08% (mean ± SD) of the feed offered (on a DM basis) over the course of the experiment, and was not different across treatments (Table 1). Substituting WS and BP for AH and CS in the diets with similar forage uNDF_30_ resulted in comparable ECM production (42.2 ± 0.99 kg/d), without any adverse effect on DMI. In the companion paper, the detailed description of ingredients and chemical composition of the experimental diets as well as particle size distribution were reported (Kahyani et al., 2019). Briefly, the proportions (% of DM) of CS, AH, BP, and WS were 22.0, 18.0, 2.0, and 0.0 in the **WS0** diet; 12.7, 7.08, 7.60, and 11.2 in the **WS50** diet; and 0.0, 0.0, 12.4, and 22.3 in the **WS100** diet, respectively. Chop length of CS at harvest was 25 to 30 mm. Alfalfa hay and WS were chopped at the length cut of 15 and 10 mm, respectively. Wheat straw was chopped using a threshing machine that was designed to separate cereal grains from straw (Golchin Trasher Hay Co.). Twenty-four hours before feeding, the chopped, dry WS was reconstituted in airtight containers to achieve a DM content of 25%, according to the procedure explained earlier (Kahyani et al., 2019). Undigested NDF_30_ concentration (% of DM) in AH, CS, WS, and BP averaged 32.0, 37.3, 62.4, and 7.70, respectively (Kahyani et al., 2019), which was determined according to the in situ procedure described in the companion paper (Kahyani et al., 2019). Diets were formulated to have similar energy density (NE_L_ = 1.65 Mcal/kg of DM) and CP concentration (16.3 ± 0.25% of DM). Forage-to-concentrate ratio was 40:60, 31:69, and 22:78 for WS0, WS50, and WS100, respectively, but diet DM, forage uNDF_30_, and dietary NDFD_30_ contents were similar across diets, averaging 46.1% DM, 13.9% of DM, and 42.3% of NDF, respectively. Dietary starch concentration averaged 29.0, 26.8, and 23.6% of DM for WS0, WS50, and WS100, respectively (Kahyani et al., 2019).

**Table 1:** Effects of substitution of wheat straw and beet pulp for corn silage and alfalfa hay on feed consumption pattern of mid-lactation Holstein cows (n = 12)

Item	Diet^1^			SEM	*P*-value	
	WS0	WS50	WS100		Linear	Quadratic
Diurnal DMI, kg						
0–2	7.74	9.19	8.66	0.46	0.15	0.07
2–4	1.78	2.19	2.43	0.35	0.09	0.80
4–6	1.27	0.68	0.55	0.25	0.02	0.37
6–24	14.5	13.6	11.9	0.74	<0.01	0.41
Refusals, % of total feed offered (DM basis)	8.54	10.2	10.5	0.81	0.17	0.60

1 WS0 = 0% forage 30-h undigested neutral detergent fiber (uNDF_30_) from wheat straw (WS); WS50 = 50% forage uNDF_30_ from WS; WS100 = 100% forage uNDF_30_ from WS.

The delivered and remaining amounts of the diets in form of TMR were weighed and sampled daily for each cow from d 22 to 28 of each period 2, 4, 6, and 24 h after feed delivery in the morning, to determine diurnal pattern of DMI. All samples were immediately frozen at −20°C pending analysis.

On d 26 and 27 of each period, behavioral activities were monitored visually for each cow over a 24-h period using 4 trained observers. The observation was based on instantaneous recording, and scanning all 12 cows took around 1 min. Eating and ruminating activities were noted at 5-min intervals, and each activity was assumed to persist for the entire 5-min interval. The 5-min interval was selected based on the validated methodology of Endres et al. (2005), identifying a high correlation (r > 0.95) between the 1-min video observations and scan intervals of 10 min or less, confirming that the 5-min scans are capable of capturing information on feeding behavior in an efficient and adequate manner. Chewing activity was defined as the sum of eating and rumination activities (Maekawa et al., 2002). A period of rumination was defined as at least one observation of rumination occurring after at least 5 min without rumination (Maekawa et al., 2002). Feeding activity was separated into meals using a meal criterion of 30 min, which is close to the validated value of 27.7 min, as reported by DeVries et al. (2003). A meal criterion is the minimum time interval between 2 meals and is used to determine meal frequency and meal duration, and a meal consisted of eating and interval times or intervals between feeding visits within a meal (Black et al., 2016). The time spent eating, ruminating, and chewing per kilogram of DM, NDF, or uNDF_30_ intake, as well as the number of meals per day, number of ruminations per day, and eating rate (total daily DMI/total eating time; g of DM/min) were averaged by cow within the period (Kahyani et al., 2013).

On the last day of each period, rumen fluid samples (approximately 3 mL) were taken 4 h after morning feed delivery from the ventral sac via rumenocentesis (Nordlund and Garrett, 1994). A 2-mL portion was acidified with 200 µL of 25% metaphosphoric acid/mL and kept frozen at −20°C for VFA analysis. Another 1-mL portion was acidified with 200 µL of sulfuric acid (1% wt/vol)/mL and kept frozen at −20°C for ammonia-N analysis. After thawing, ruminal fluid samples for ammonia-N analysis were centrifuged at 30,000 × *g* for 20 min at 4°C. The supernatant was harvested for ammonia-N assay using the method of Broderick and Kang (1980). For VFA analysis, the acidified samples were thawed and centrifuged at 10,000 × *g* at 4°C for 20 min and analysis was undertaken using GC (0.25 × 0.32, 0.3 μm i.d. fused silica capillary, model no. CP-9002 Vulcanusweg 259 a.m., Chrompack).

Data were analyzed using Proc Mixed of SAS (2002, SAS Institute Inc.). The model included fixed effects of square, period, and treatment. Cow within square was included in the Random statement. Normality of distribution and homogeneity of variance for the residuals were tested using Proc Univariate (SAS Institute Inc.). If the Shapiro-Wilk value (W) was 0.98 or greater, the variables were considered normal. The differences among the treatments were evaluated using the Tukey-Kramer method. Polynomial orthogonal contrasts were used to identify the linear and quadratic effects. Significance was declared at *P* ≤ 0.05, and tendencies were noted if 0.05 < *P* ≤ 0.10.

Data of DMI during the day are reported in Table 1. From 0 to 2 h, there was a tendency observed for a quadratic effect (*P* = 0.07) on DMI, with cows fed WS50 consuming the greatest amount of DM (9.19 kg). From 2 to 4 h after feeding, there was a tendency observed for a linear increase (*P* = 0.09) on DMI as WS and BP increased. Later DMI (4 to 6 h and 6 to 24 h after feeding) decreased linearly as WS and BP increased.

Data of chewing activities and meal patterns are presented in Table 2. There was a linear decrease in time spent for eating (*P* = 0.01) and total chewing (*P* = 0.04) as the dietary proportion of WS and BP increased. Rumination time was not different across treatments, averaging 455 ± 21.2 min/d (*P* = 0.22). Eating and chewing time expressed per kilogram of DM, NDF, and uNDF_30_ decreased linearly as WS and BP inclusion increased in the diets. The incremental inclusion of WS and BP in the diets resulted in a linear decrease in number of meals from 10.3 to 8.8 meals/d (*P* = 0.01), and a linear increase in time between meals, eating rate, as well as meal size per kilogram of DM (from 2.49 to 3.02; *P* < 0.01). The rumination patterns were not different across treatments, but the number of bouts/day was the greatest in WS50-fed cows, averaging 13.0 bouts/d (*P*_Quadratic_ = 0.06).

**Table 2:** Effects of substitution of wheat straw and beet pulp for corn silage and alfalfa hay on chewing activities and meal patterns of mid-lactation Holstein cows (n = 12)

Item	Diet^1^			SEM	*P*-value	
	WS0	WS50	WS100		Linear	Quadratic
Eating						
Min/d	303	300	272	10.0	<0.01	0.10
Min/kg of DM	12.3	12.0	11.2	0.61	0.02	0.40
Min/kg of NDF	39.3	37.8	35.0	1.95	<0.01	0.56
Min/kg uNDF_30_	68.0	66.4	58.3	2.75	<0.01	0.10
Rumination						
Min/d	461	473	432	21.2	0.22	0.19
Min/kg of DM	18.8	19.4	18.0	1.14	0.41	0.24
Min/kg of NDF	60.3	61.1	56.2	3.66	0.20	0.30
Min/kg uNDF_30_	104.1	107.1	93.8	6.30	0.08	0.14
Total chewing						
Min/d	764	773	704	21.9	0.01	0.04
Min/kg of DM	31.1	31.5	29.2	1.45	0.05	0.10
Min/kg of NDF	99.6	98.9	91.2	4.66	0.01	0.17
Min/kg uNDF_30_	177	166	155	7.34	<0.01	0.93
Meal pattern						
Eating						
Number of meals/d	10.3	9.5	8.8	0.53	0.01	0.90
Length, min/meal	27.1	30.2	29.8	2.50	0.18	0.32
Time between meals, min	109	129	134	7.51	0.01	0.38
Rate, g of DM/min	84.2	85.2	90.2	4.17	0.04	0.40
Meal size, kg of DM	2.5	2.7	3.0	0.15	<0.01	0.94
Rumination						
Bouts/d	12.2	13.0	11.3	0.55	0.28	0.06
Min/bout	38.6	36.9	39.4	1.62	0.73	0.27
Time between bouts, min	72.8	71.5	81.1	5.54	0.29	0.41
Rate, g of DM/min	56.3	55.4	58.6	3.51	0.38	0.35

1 WS0 = 0% forage 30-h undigested neutral detergent fiber (uNDF_30_) from wheat straw (WS); WS50 = 50% forage uNDF_30_ from WS; WS100 = 100% forage uNDF_30_ from WS.

The incremental addition of WS and BP to the diets resulted in a linear decrease in propionate concentrations but linear increases in acetate and ruminal ammonia-N concentrations (*P* = 0.01; Table 3).

**Table 3:** Effects of substitution of wheat straw and beet pulp for corn silage and alfalfa hay on ruminal fermentation of mid-lactation Holstein cows (n = 12)

Item	Diet^1^			SEM	*P*-value	
	WS0	WS50	WS100		Linear	Quadratic
Total VFA, m*M*	108	112	91.9	5.58	0.06	0.08
Individual VFA, m*M*/100 m*M*						
Acetate	68.3	67.8	71.6	1.07	0.05	0.12
Propionate	22.8	21.7	19.6	0.73	0.01	0.34
Butyrate	6.88	7.60	6.41	0.41	0.42	0.06
Isobutyrate	0.38	0.42	0.36	0.04	0.72	0.27
Valerate	0.85	0.93	0.64	0.11	0.15	0.13
Isovalerate	1.33	1.53	1.42	0.10	0.58	0.26
Acetate:propionate	3.14	3.21	3.68	0.14	0.01	0.24
Ammonia-N, mg/dL	10.1	10.4	12.4	0.76	0.01	0.25

1 WS0 = 0% forage 30-h undigested neutral detergent fiber (uNDF_30_) from WS; WS50 = 50% forage uNDF_30_ from WS; WS100 = 100% forage uNDF_30_ from WS.

The present study investigated the effects of diets containing different forage qualities but formulated to supply similar levels of forage uNDF_30_ and dietary NDFD_30_ on diurnal feed intake patterns, feeding behavior, and ruminal fermentation of high-producing dairy cows. Diets containing WS and BP had a lower proportion of long particles (particles >19 mm; Kahyani et al., 2019) than WS0, which is indicative of the alteration in the physical characteristics of diets with increasing WS and BP inclusion. Particles retained on the 19-mm sieve are more effective in stimulating chewing than particles retained on the 8-mm sieve (Heinrichs and Kononoff, 2002), possibly explaining the lower chewing activity in cows fed WS100 as this diet contained less particles retained on the 19-mm sieve (Kahyani et al., 2019).

The incremental incorporation of WS and BP in the diets (WS50 and WS100) resulted in the lower concentration of uNDF_288_ in these diets, helping to explain why cows consuming these diets spent less time for eating and chewing. Intake of uNDF_288_ decreased linearly from 2.48 to 2.07 kg/d (Kahyani et al., 2019) as WS and BP level increased in the diet. The increased chewing activity facilitates the escape of fiber from the rumen mainly because of size reduction and increased digestion rate. As dietary uNDF_288_ concentration increases, a greater proportion of the fiber is reliant upon chewing action for passage (Smith, 2019). Therefore, the decrease in chewing time observed in WS-containing diets could be related to the lesser intake of uNDF_288_ in these cows that likely required less need for particle size reduction (Beauchemin et al., 2001). In addition, lower forage-to-concentrate ratio in WS-containing diets (Kahyani et al., 2019) may also help to explain the linear reduction in chewing time as feeding the denser diets (higher concentrate proportion) is usually associated with less chewing activity in dairy cows (Farmer et al., 2014; Omidi-Mirzaee et al., 2017).

Experimental diets had no effect on rumination activity despite the differences in particle size distribution among treatments (Kahyani et al., 2019). The differences in particle size intakes are known to influence rumination activity in dairy cows (Mertens, 1997; Nasrollahi et al., 2016), suggesting the greater importance of the physicochemical properties of forage component (i.e., similar forage uNDF_30_ content) than the particle size intakes. These findings support our initial hypothesis that diets containing forages of various qualities but with similar forage uNDF_30_ and dietary NDFD_30_ will have a similar rumination response. Jones and Siciliano-Jones (2015) identified a close link between uNDF_30_ concentration and the amount of forage retained in the rumen, which could potentially influence rumen motility and induce rumination.

Lower molar concentration of propionate in cows consuming the WS-containing diets could be explained by the lesser intake of starch in these diets (Kahyani et al., 2019) because starch fermentation in the rumen results in propionate production (Grant and Ferraretto, 2018; Smith, 2021). Pectin makes up a large portion of the NFC in BP, and ruminal fermentation of pectin substances produces more acetate and butyrate and less propionate (Munnich et al., 2017), possibly providing evidence for the greater acetate-to-propionate ratio with the incremental increase in BP inclusion in the diets.

In the companion paper (Kahyani et al., 2019), the increased inclusion of WS and BP in the diet resulted in a linear increase in milk fat content, which is supported by a linear increase (*P* = 0.05) in acetate molar concentration (Table 3). The replacement of slowly fermentable carbohydrates with rapidly fermentable carbohydrates causes the acetate-to-propionate ratio to decrease, a factor contributing to milk fat reduction in dairy cows (Zebeli et al., 2006). The WS0 diet had a higher proportion of forage-to-concentrate with greater concentration of starch and NFC (Kahyani et al., 2019). The greater fermentability of the carbohydrate component in WS0 compared with WS50 and WS100 diets is possibly the main contributor to the lower acetate-to-propionate ratio in the rumen, and thus milk fat depression.

Beet pulp and WS substitution for CS and AH resulted in increase of ammonia-N concentration in the rumen, which is suggestive of the less efficient protein utilization, and a better synchronization of energy and nitrogen for microbial protein synthesis in WS0-fed cows. Increased availability of fermentable carbohydrates improves the utilization of ammonia in the rumen (Hristov et al., 2005). The greater starch intake in WS0 than in WS50 and WS100 (Kahyani et al., 2019) is linked to the increased availability of fermentable carbohydrates in the rumen, which possibly improved the microbial utilization of dietary N and decreased ammonia accumulation in the rumen. Eastridge et al. (2009) reported that nitrogen efficiency expressed as milk urea nitrogen or milk protein was not different between cows fed diets containing straw but formulated to supply similar concentration of NFC.

Overall, the incorporation of WS and BP in place of AH and CS in dairy cow rations with fixed forage uNDF_30_ and dietary NDFD_30_ levels had no negative effects on total feed intake and rumination activity but the substitution increased ruminal pH and acetate-to-propionate ratio. These beneficial effects could have resulted from a good combination of WS and BP, and possibly the role of WS fiber in maintaining rumination activity and increasing ruminal pH. Therefore, BP could be considered as an alternative to high-quality forages when WS was added to maintain uNDF_30_. However, feeding WS as the sole forage source increased ruminal ammonia-N concentration while decreasing eating and chewing time. Further research on the supplementation of readily available sources of carbohydrates or degradable proteins at the ruminal level with WS-containing diets is required to optimize nitrogen efficiency.
